# HIV-1: To Splice or Not to Splice, That Is the Question

**DOI:** 10.3390/v13020181

**Published:** 2021-01-26

**Authors:** Ann Emery, Ronald Swanstrom

**Affiliations:** 1 Lineberger Comprehensive Cancer Center, University of North Carolina, Chapel Hill, NC 27599, USA; aemery@email.unc.edu; 2 Department of Biochemistry and Biophysics, University of North Carolina, Chapel Hill, NC 27599, USA; 3 Center for AIDS Research, University of North Carolina, Chapel Hill, NC 27599, USA

**Keywords:** HIV-1, HIV-1 splicing, HIV-1 oversplicing, HIV-1 latency

## Abstract

The transcription of the HIV-1 provirus results in only one type of transcript—full length genomic RNA. To make the mRNA transcripts for the accessory proteins Tat and Rev, the genomic RNA must completely splice. The mRNA transcripts for Vif, Vpr, and Env must undergo splicing but not completely. Genomic RNA (which also functions as mRNA for the Gag and Gag/Pro/Pol precursor polyproteins) must not splice at all. HIV-1 can tolerate a surprising range in the relative abundance of individual transcript types, and a surprising amount of aberrant and even odd splicing; however, it must not over-splice, which results in the loss of full-length genomic RNA and has a dramatic fitness cost. Cells typically do not tolerate unspliced/incompletely spliced transcripts, so HIV-1 must circumvent this cell policing mechanism to allow some splicing while suppressing most. Splicing is controlled by RNA secondary structure, cis-acting regulatory sequences which bind splicing factors, and the viral protein Rev. There is still much work to be done to clarify the combinatorial effects of these splicing regulators. These control mechanisms represent attractive targets to induce over-splicing as an antiviral strategy. Finally, splicing has been implicated in latency, but to date there is little supporting evidence for such a mechanism. In this review we apply what is known of cellular splicing to understand splicing in HIV-1, and present data from our newer and more sensitive deep sequencing assays quantifying the different HIV-1 transcript types.

## 1. How Splicing Works Generally for Cellular mRNAs and for HIV-1

### 1.1. HIV-1 Splicing Overview

**Figure 1:**
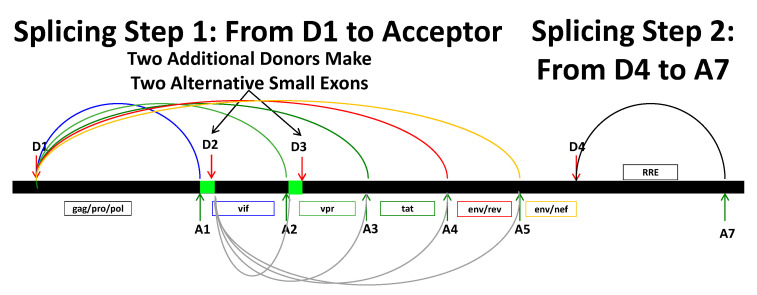
Splicing in HIV-1. Most HIV-1 transcripts remain unspliced. When splicing occurs, it always splices from D1 to one of the downstream acceptors, removing a section under a colored arc. Once having spliced from D1, the RNA may splice again to remove the sequence between D4 and A7 containing the *vpu*/*env* genes and the RRE (Rev Response Element; the region removed is sometimes called the *env* intron). Transcripts that undergo this additional D4 to A7 splice (needed to create *tat*, *rev*, and *nef* mRNAs, and short mRNAs for *vif* and *vpr*) comprise the group of fully spliced transcripts. Transcripts that splice only from D1 but not from D4 to A7 (*vif*, *vpr*, *vpu*/*env* mRNAs, and a long transcript isoform of *tat* mRNA) make up the partially/incompletely spliced transcripts. Two small exons (shown in green) can be included or skipped in mRNAs other than *vif*. Possible splice patterns that include the first small exon are shown when D1 splices to A1 and then splicing occurs from D2 to a downstream acceptor (shown by the gray arcs below the line). The second small exon can be included by a similar mechanism when splicing from D1 to A2 (or D2 to A2) is followed by splicing from D3 (not shown).

RNA synthesis from the HIV-1 provirus results in only full-length transcripts, and most avoid splicing to remain full length at approximately 9.2 kb [1,2,3]. These unspliced RNAs are the mRNAs encoding the *gag*/*pro*/*pol* genes and function as the genomic RNA that is packaged into new virions. All other viral RNA products are the result of splicing (Figure 1). Transcripts that splice always use splice donor D1 in the 5′ UTR and splice to one of the downstream acceptors A1 through A5. The area under the arc gets spliced out and the sequence upstream of D1 is juxtaposed to the next ORF downstream of whichever acceptor site was used. Thus, splices to A1 make a *vif* transcript, to A2 a *vpr* transcript, and so on. This is the first step of splicing.

Once splicing from D1 has occurred, the transcript may or may not splice out the sequence from D4 to A7. D4 to A7 contains the coding sequences for *vpu* and *env*, and the Rev Response Element (RRE). This D4 to A7 splice happens only if D1 has already been used. Transcripts that splice from D1 but do not splice out D4 to A7 are longer and are collectively called the 4 kb size class or partially/incompletely spliced, since they retain the “*env*” intron. Those RNAs that splice from D1 and splice out the D4-A7 segment are the shorter 1.8 kb size class of viral mRNAs, also called completely spliced. In general, cellular transcripts that are not completely spliced are not exported from the nucleus and so HIV-1 unspliced and partially spliced transcripts (which retain the D4-A7 sequence with the RRE) cannot be exported by the normal cellular mRNA export pathway [4]. To circumvent this restriction, the viral Rev protein binds the RRE’s complex secondary structure (in the retained *env* intron) and links the unspliced/partially spliced transcript to the alternate CRM1-mediated nuclear export pathway. It is also possible, though infrequent, to splice directly from D1 to A7, which creates a transcript isoform of *nef* mRNA.

An unusual feature of HIV-1 splicing is the optional inclusion of two small noncoding exons. Two additional splice donors, D2 and D3, can be used to make these small exons, shown in green in Figure 1. Either or both small exons can be included in a transcript, with the example in Figure 1 for inclusion of the A1-D2 small exon. Collectively these permutations of unspliced, partially spliced and completely spliced, combined with either, both, or none of the small exons give rise to over 50 well characterized final viral RNA transcripts.

Generally, splicing is a fast and efficient cotranscriptional process but in HIV-1 it must be highly suppressed to make full length or partially spliced transcripts. In these cases, introns are retained, which is something that typically does not happen with cellular mRNA transcripts. Many studies have asked “what controls the inclusion or skipping of the small exons”, and “how does splicing from D1 modulate acceptor site usage between A1, A2, A3, A4, and A5 so that enough of everything is made” (See [5] for a review).

### 1.2. Cellular mRNA Splicing Basics

Understanding HIV-1 splicing must be based in an understanding of cellular splicing in general. A typical cellular transcript has short coding sequences (exons, meaning “expressed”) interspersed with longer non-coding sequences (introns, meaning “intervening”). Exons are usually between 100–200 nucleotides long. Introns tend to be between 1000–2000 nucleotides, though in some instances they are much longer. The 5′ end of an intron is the donor (or 5′ splice site) and the 3′ end is the acceptor (or 3′ splice site). Splicing removes the intervening intronic sequence and joins the donor to the acceptor. As a gene is transcribed it is processed into a mature mRNA by attaching a 5′ cap, splicing out all the intronic sequences and joining the exons, and adding a poly A tail at the 3′ end.

Spliceosome components recognize exons based on their paired acceptor and donor sites and form early splicing complexes that span the exon. This is called “exon definition” and is essential for understanding how HIV-1 splicing works [6]. Consensus splicing signals at each exon end identify paired donor and acceptor sites spaced at a typical exon length. These potential exon signals are recognized initially by the cellular small nuclear riboproteins snRNP U1 and snRNP U2. A consensus sequence at the 5′ donor site is recognized and bound by U1, while for the 3′ acceptor site, U2 recognizes and binds to a consensus sequence and a branch point/poly pyrimidine tract about 25–30 nucleotides upstream of the acceptor. Spliceosome elements recognize these splicing signal sequences and assemble at both ends of the exon, and this defines the exon and targets it for inclusion in the spliced transcript [7]. In some as of yet incompletely understood process, the defined exons are brought together and the catalytic events of splicing take place resulting in a spliced transcript with all the introns removed (for an intriguing possibility see [8]). Note that the donor from one exon is joined to the acceptor of the next downstream exon—the splice sites that must be recognized to define an exon are not themselves joined together as donor and acceptor.

Many false pseudo-consensus splice signal sites exist in pairs spaced across an exon-sized sequence, but such randomly occurring potential splice sites are seldom used [9]. An additional layer of control is needed to correctly identify genuine exons, and this is accomplished by cis-acting splicing regulatory elements (SRE). SREs are short sequence elements, usually from 5 to 20 nucleotides long, that bind trans-acting cellular proteins and identify or mask splice sites. SREs are named by their functions and locations. Elements that enhance splicing are called Exonic Splicing Enhancers (ESE) if contained in an exon, or Intronic Splicing Enhancers (ISE) if in an intron. Elements that suppress splicing are Exonic Splicing Silencers (ESS) or Intronic Splicing Silencers (ISS) [10].

**Figure 2:**
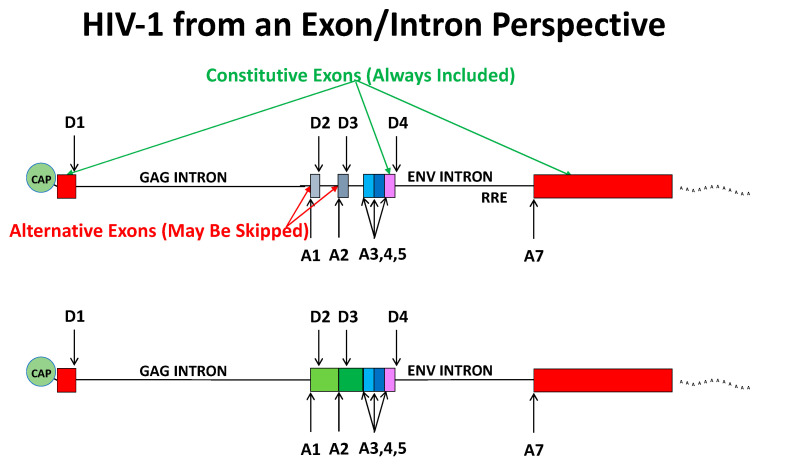
Exons in HIV-1. (**Upper Panel**) The first exon is defined by the cap and D1. The final exon is defined by A7 and the poly A site. The two small alternative exons are defined by A1 and D2, and A2 and D3 (grey). The exon defined by D4 is constitutive, but can use A3, A4, or A5 for the acceptor to define the exon. (**Lower Panel**) D4 can also use A1 or A2 to define the *vif* or *vpr* exons respectively, independently of D2 and D3.

The first and last exons are special cases [11]. The first exon is defined by the 5′ cap and U1 binding at a downstream donor [12]. The last exon is defined by the final acceptor and the poly A site, and poly A processing requires an upstream acceptor, but not necessarily splicing to that upstream acceptor [13]. In HIV-1 the first exon spans the transcription start to the first splice donor, D1. The final exon starts at A7 and ends at the poly A tail (Figure 2). The first and last exons are always included.

An internal exon that is always included (a “constitutive” exon) has the splice acceptor and donor signal sequences at each end of the exon. All exons have an enhancer element (ESE) somewhere in the exon. This enhancer is bound by one of the family of SR proteins, which recruits and stabilizes the formation of the early spliceosome spanning the exon. This process defines the exon and ensures it will be included in the transcript.

In addition to constitutive exons, there are also “alternative” exons that are sometimes skipped. If an exon does not get defined, then it is considered part of an intron and it gets spliced out. Like constitutive exons, an alternative exon also has the splicing signal sequences at the exon boundaries, but it has two splicing regulatory elements—an exonic splicing enhancer (ESE) but also an exonic splicing silencer (ESS). Typically, these two regulatory elements are near each other in the sequence. The splicing outcome depends on what happens first: if an SR protein binds to the enhancer then the spliceosome begins to assemble, the exon is defined, and it gets included. But if an hnRNP protein binds to the silencer then spliceosome assembly is blocked, the exon is not recognized, and it gets skipped. Thus, in every exon we would expect to find at least one enhancer, and in every alternative exon we also expect to find a silencer [14,15].

While it is not the purpose here to give a detailed explanation of the catalytic splicing mechanism, it is critical to understand that the recognition and binding of the small nuclear riboproteins U1 and U2 at the donor and acceptor sites respectively are the first steps to defining an exon, and that HIV-1 splicing is best understood using the exon definition model [5]. Binding of U1 to the donor is the first step in defining the exon but it does not always imply splicing from that donor. U1 can disengage without splicing, and in any case, that donor cannot splice unless another downstream exon is defined for it to splice to [16,17,18].

HIV-1 has 5 exons and splicing of viral RNA follows the rules of exon definition (Figure 2, top). The first and last exons are always used. The first small exon is defined by A1 and D2. The second small exon is defined by A2 and D3. Alternative exons may be skipped, and this is what usually happens with the two small exons—most of the time they do not get defined and are left out. Some exons have more than one possible acceptor site, and this is the case with the exon defined by D4, which is always included (except in the rare D1-A7 splice product) but may use any of the upstream acceptors. Depending on where the spliceosome components bind, one of multiple acceptor choices is made.

These basic rules of exon definition explain most features of HIV-1 splicing, but exactly how *vif* (A1) and *vpr* (A2) transcripts are made is not obvious. These transcripts splice from D1 to A1 or A2 respectively. Most transcripts that splice to A1 or A2 splice again from D2 or D3, creating a small exon. However, for a *vif* or *vpr* transcript, D2 and D3 are not used. A mechanism that defines the small exon for acceptor usage but then does not use the downstream donor has not been well defined for HIV-1. One possibility is that U1 engages with D2 (or D3) to define A1 (or A2) and then that U1 disengages [19]. Another possibility is that exon definition spans from A1 to D4 (or A2 to D4) to define the *vif* (or *vpr*) transcripts (Figure 2, bottom). Such a *vif* exon is about 1100 nucleotides—long for an exon, but not impossibly long. There are short *vif* and *vpr* isoforms that also splice from D4 to A7—good evidence of D4 defining the upstream exon and then splicing from D4 to the terminal exon.

There is also good evidence that D2/D3 are not required for *vif*/*vpr* transcripts. A study of splicing in transmitted/founder viruses found that differences in D3 sequence caused a wide range of splicing to A2 (*vpr*) but did not increase or reduce *vpr* transcripts proportionally. In the case of one isolate with a defective D3 and very little splicing to A2, the percentage of *vpr* transcripts actually increased, while the percentage of the A2 to D3 small exon decreased. This suggests that D3 is not essential to defining A2 for *vpr* transcripts, which seem to be produced independently from the efficiency of D3. Conversely, mutations to an SRE that upregulated splicing to A2 greatly increased the percentage of transcripts that contained the A2-D3 small exon, but this also did not proportionally increase the percentage of *vpr* transcripts.

There is similar interesting data with *vif* transcripts. We mutated a splicing silencer in the A1-D2 small exon. This would be expected to increase A1 usage, which it did, but not to impact D2 which is some distance away. We saw an increase in *vif* transcripts but a decrease in transcripts containing the A1-D2 small exon. A mutation to a different splicing enhancer M1, which is at D2, reduced usage of A1, as expected, but greatly reduced the proportion of those A1 transcripts with the small A1-D2 exon in favor of *vif* transcripts [20].

Though these observations represent a limited sample set, these examples suggest that the A1 (*vif*) and A2 (*vpr*) exons may be defined by D4, leaving D2 and D3 to define the small exons. This revisits a long-standing question about the purpose of these small exons. It is unlikely in the economy of a rapidly mutating retrovirus that they exist solely to define themselves. On the other hand, diligent investigation into the purpose of the small exons has failed to find any benefits for translation efficiency or stability in mRNAs with or without these small exons [5]. Perhaps D2 and D3 define the small exons in competition with D4, so that *vif* and *vpr* represent a lesser percentage of total spliced transcripts.

So far it has been shown how HIV-1 follows the exon definition rules for cellular splicing but this applies only to fully spliced transcripts. It is interesting that in most cellular transcripts, all of the introns are spliced out [21], but in HIV-1, the rules are broken—introns are retained and splicing is so heavily suppressed that most transcripts do not splice at all.

## 2. Complete HIV-1 Splicing

Complete splicing is the default behavior for cellular mRNAs, so some completely spliced (1.8 kb) HIV-1 RNAs are always made. These fully spliced mRNAs include *tat*, *rev*, and *nef* transcripts. This suggests that targeting splicing to reduce *tat* or *rev* mRNAs is unlikely to be successful. Their production requires nothing special from the virus—they are the expected outcome of normal cellular processes. To alter their production would require altering cellular splicing.

## 3. HIV-1 with No Splicing at All

On the other hand, unspliced HIV-1 transcripts exist in defiance of cellular splicing rules. This retention of introns depends on sequences in the viral genome and thus is a virus-specific suppression of splicing that could be an attractive therapeutic target. The obvious question is “how does HIV-1 manage to keep most of the transcripts completely unspliced?” One interesting possibility is the structural masking of the D1 splice site to prevent splicing from ever starting. It has been noted that the 5′ UTR contains a number of highly structured functional regions, and that multiple structural configurations exist [22,23]. It was recently shown that an additional one or two G’s at the transcription start site favors one of two 5′ UTR secondary structures. The structure with a single G promotes dimerization and stabilization of the dimerized form of viral RNA and sequesters D1, while the other structure (with two or three G’s) leaves D1 accessible. These alternate structures exist in equilibrium. Splicing is favored by the conformation that exposes D1 and reduced in the conformation that occludes it [24]. This structural blocking of D1 may be sufficient to prevent splicing from D1 in the majority of transcripts.

Blocking D1 usage appears to be sufficient to eliminate all splicing, including splicing from the other downstream donors D2, D3, and D4. In the absence of D1 usage, none of the other donors are used either [25,26,27]. It has been observed that increasing the length of the first exon decreases splicing efficiency [28]. Analysis of first exons found that the mean length was 149 nucleotides, and the maximum length was 1316 nucleotides [29,30]. From the 5′ cap to D1 in HIV-1 is less than 300 nucleotides, but in the absence of D1 the first exon would span the transcription start site to D2, which is longer than 4000 nucleotides. The failure to splice when D1 is occluded may be the failure to produce an acceptable first exon. It may therefore be that blocking splicing at D1 is sufficient to shut off all splicing.

An unexpected observation that has been reported is that HIV-1 infection of primary T cells causes an increase in intron retention in cellular genes [31]. This raised the question of whether a viral gene product is responsible for HIV-1 splicing suppression, and if so, could this virus-specific mechanism spill over into some cellular splicing suppression. We found that very little intron retention in cellular transcripts occurs in 293T cells transfected with an infectious HIV-1 plasmid, and that it was not targeted to specific transcripts, as would be expected for a Rev-mediated effect [32]. Another possible explanation is that HIV-1 infection triggers an immune related response or general cellular distress in the primary T cells that impacts cellular splicing more generally.

## 4. Oversplicing—When HIV-1 Splicing Gets Out of Control

Sometimes splicing suppression is lost and the percentage of unspliced transcripts is reduced. This is known as oversplicing and is the one splicing perturbation that has an impactful effect on viral fitness through the loss of Gag and Gag-Pro-Pol precursor polyproteins [5,33,34]. Several things can cause oversplicing. All alternate exons have at least one exonic splicing suppressor (ESS) that inhibits use of that exon’s acceptor. When these suppressor elements are mutated, suppression to that specific acceptor is lost and transcripts splice excessively to that acceptor, causing a decrease in unspliced transcripts. Beyond the known ESS elements in the HIV-1 genome, there are likely more to be found [33]. Sometimes the loss of a single suppressor is sufficient to cause oversplicing. Some suppressors work synergistically [34]. This contradicts a previously held idea that splicing suppression was caused by weak acceptors [35,36,37], but oversplicing shows that in the absence of a specific suppressor, one acceptor becomes very strong indeed [34]. In contrast, D2 and D3 have been considered weak donors, and as such may contribute to splicing suppression through poor exon definition [38,39]. Studies have shown that when D2 and D3 are mutated to more closely match the consensus U1 recognition sequence (or when U1 is adapted to more closely match D2 or D3), oversplicing to A1 and A2 respectively occurs [39,40,41]. Both the loss of suppressors and enhancement of the downstream donor likely work by increasing exon definition of otherwise alternative exons.

In addition to the silencer elements that suppress splicing at the canonical splice sites, a silencer has been found that suppresses oversplicing to a little used aberrant splice site, D2b [42]. D2b is one of several seldom used but conserved ‘mis’-splicing sites that requires suppression to prevent exon definition and oversplicing. Other similarly conserved but infrequently used HIV-1 splice sites exist but have yet to be studied, and it will be interesting to see if they are kept under control with suppressor elements, as they are in cellular transcripts [43]. This suggests that unwanted splice sites are created in the genome because of other sequence requirements (e.g., specific amino acid codons) and must be permanently suppressed. Oversplicing has also been triggered by overexpression of SR proteins [44] and by digoxin [45].

**Figure 3:**
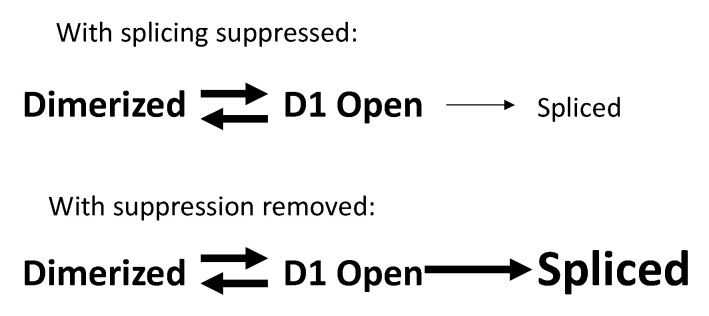
A possible model for oversplicing when a distant suppressor is lost. The 5′ UTR can fold into one of two conformations that exist in equilibrium—dimerized or with D1 open for splicing. (**Top**) If splicing is suppressed, then a small proportion of the transcripts exposing D1 will splice. (**Bottom**) If a suppressor is removed, almost all transcripts with open D1 will splice to an unsuppressed acceptor. This drives the dimerized/D1 open equilibrium to the right and the unspliced (dimerized) full-length transcripts are lost.

The equilibrium between the two alternative 5′ UTR structures mentioned above [24,46,47] and their differential tendencies to splice suggests a model for how loss of distant suppressor elements might cause oversplicing from D1. The 5′ UTR structures are in a dynamic equilibrium, each favoring a specific folding, but able to occasionally adopt the other conformation. The transcripts that expose D1 begin to splice co-transcriptionally (Figure 3, top). When the suppressors are functional, D1 OPEN to SPLICED is relatively slow, and by the time transcription finishes and the mRNA is exported, most of the RNA is still unspliced (Figure 3, top). When a suppressor is lost, D1 OPEN to SPLICED is rapid to that unsuppressed acceptor (Figure 3, bottom). This drives the equilibrium toward D1 OPEN and by the time transcription has finished, most transcripts will have spliced. Sequences downstream of D1 are required for the stable dimerized 5′ UTR structure, so their removal by splicing is a one-way trip.

We wondered what would happen when multiple silencers were mutated. When there is no suppression of either A2 or A3, we asked which would be used. Almost all of the transcripts are one of two types, and they all splice to both A2 and A3. Most transcripts splice from D1 to A2 then again from D3 to A3 (with the small A2-D3 exon). Some transcripts splice first to A1, then to A2, and finally to A3. This strongly suggests that HIV-1 splicing happens in a sequential manner, based on which acceptor is transcribed first. A1 is transcribed first and some transcripts splice to it in a normal proportion. Then once unsuppressed A2 is available, everything splices to it. Finally, when the unsuppressed A3 is available, everything again splices to it. We did not see any direct splicing from D1 to A3, hinting that A3 is not available for splicing until after A2 [34]. This is compelling evidence of sequential cotranscriptional splicing and reinforces the extent to which HIV-1 uses cis-acting sequences to suppress most splicing events. Because oversplicing of HIV-1 has such a dramatic fitness cost, it may be a significant Achilles heel of splicing and a potentially important therapeutic target [41].

## 5. Partially Spliced HIV-1: Both Splicing and Not Splicing

Partially spliced transcripts splice at D1 but not at D4. Like unspliced transcripts, they are dependent on Rev for nuclear export. They may also be dependent on Rev for splicing suppression. D4 is a strong donor and will always be used for exon definition. The final exon from A7 to the poly A site must be defined and included or there would be no poly A processing. The question then is how splicing is blocked from D4 to A7 in these partially spliced RNAs.

The suppressors of splicing at D4 and A7 are not as well defined as they are for other splice sites. To be fair, it is an incredibly challenging problem because there are multiple open reading frames at both ends of the D4-A7 intron that complicate mutagenesis. Previous work has used artificial constructs that likely incompletely represent HIV-1 splicing. Multiple elements are proposed to exist, and yet generally they are broadly defined and have minimal or inconsistent effects [48,49,50]. Our splicing assays have failed to corroborate previous results for some of these described SREs but instead show little effect in regulating D4 to A7 splicing when these elements are mutated [34,51]. Much more work is needed to establish the SREs that control splicing at D4 and A7.

Some interesting studies suggest that splicing suppression from D4 to A7 is primarily due to Rev rather than inefficient or blocked splice sites. It was found that Rev and CRM1 assemble co-transcriptionally on RRE-containing transcripts and remain stably associated [52]. Rev multimerizes on the RRE and hnRNP A1 multimerizes along A7. Do they synergistically compete with splicing to A7? Competition between Rev-mediated export and D4-A7 splicing has been proposed [53]. It has been shown that Rev interferes with splicing [54], and that Rev and early spliceosome assembly are mutually exclusive [55].

The potential for Rev to regulate splicing is a question that needs additional study. A competition between Rev and spliceosome formation could be mediated through several mechanisms. Perhaps the RRE needs to be transcribed quickly to form an optimal RRE structure—slower transcription may promote folding to a more proximal, less branched structure that is sub-optimal for Rev binding. We know that the RRE has multiple possible conformations [56,57]. Perhaps initial Rev binding to the RRE stabilizes the secondary structure and holds it in place until transcription is finished, while insufficient Rev lets splicing proceed.

Our data shows that the proportions of fully spliced and partially spliced viral RNAs do not vary much under any experimental conditions tested thus far, except when Rev is absent [34,51,58,59]). Increasing Rev has been previously found to decrease fully spliced mRNAs [60] while the absence of Rev increases fully spliced mRNAs or else leads to the rapid degradation of intron-containing mRNAs [61]. We have found that in a ∆Rev mutant, splicing is complete [58] and this is the only condition where we have seen a markedly skewed balance of splicing from D4 to A7. Interestingly, silencer mutations that induce extreme oversplicing from D1 to an acceptor upstream of D4 do not affect the extent of splicing from D4 to A7. Taken together, these observations suggest that splicing from D4 to A7 may be more under the control of Rev than of regulatory elements and exon definition.

The imperturbability of D4 to A7 splicing may be due to the nature of Rev self-regulation: low Rev -> everything gets spliced -> more *rev* transcripts are made -> more Rev is made -> fewer fully spliced *rev* transcripts are made -> Rev amounts decrease. This balance also suggests that *rev* transcripts could not stochastically fall below some level needed to prevent latency, since the obvious effect of low Rev is to create an abundance of *rev* mRNA transcripts.

## 6. Aberrant and Odd HIV-1 Splicing

HIV-1 splicing is sloppy [2,3,62]. The known and expected acceptors and donors are used most of the time, but a low level of inaccurate splicing can be detected across the entire genome. There are many near misses, and in particular many minor acceptor sites that are some multiple of three nucleotides from the canonical sites. This curious in-frame constraint has also been observed in cellular mRNA processing [63,64]. Overall, about 0.2 percent of spliced transcripts splice to a noncanonical acceptor near an expected acceptor site, though it’s as high as 0.8 percent for splices near A1 [3]. There are several seldom used donors and acceptors and small exons that are consistent across HIV-1 strains. An occasional strain (such as 89.6) has an additional acceptor in the *env* intron [2].

D1 and D4 can splice to downstream cellular exons of readthrough transcripts [31]. Some chimeric splicing may impact the clonal expansion of an infected cell when upregulating certain genes [65]. Trans-splicing from a downstream donor to an upstream acceptor on another transcript has also been observed, possibly the result of co-transcriptional splicing between consecutive transcripts [3]. Most alternative splicing is functionally irrelevant and at best produces a small ORF with an early stop codon. Occasionally, odd strain-specific splicing results in some idiosyncratic hybrid HIV-1 proteins of unknown functionality and importance [2,66,67]. There is a long-standing HIV-1 splicing story that blocking the usage of a canonical acceptor will activate cryptic splice sites. In our own work we have never observed this except in the case of mutations near D1 which do activate cryptic splicing [33,68], confirming an earlier report of this same D1- specific behavior [1].

It is equally interesting to see what splicing does not occur. There is little splicing from D1 directly to A7. This may reflect a general splicing rule restricting the joining of the terminal exons. There are also no transcripts including the small exons and splicing from them to A7. Such small exon–A7 splices seem possible, so perhaps they occur too rarely even to be detected by our deep sequencing assays. One would expect that an additional defined exon from A1 to D3 could exist, as an alternate form of *vif* transcript, but this has also not been observed.

## 7. HIV-1 Splicing Regulation: It’s Even More Complicated Than We Thought

Ongoing work suggests that merely binding regulatory sequences with enhancing (SR) or suppressing (hnRNP) proteins is by itself still an overly simplistic model of splicing control. (See [33] for a map of HIV-1 SREs, and [5] for an overview of HIV-1 SRE function.) Many of the published studies on HIV-1 regulatory elements propose specific SR and hnRNP proteins that regulate those elements. We saw that knock down of cellular factors reported to control HIV-1 splicing elements did not have the expected effects on the spliced RNAs produced [59]. In general, effects on splicing were small or negligible. Also, knockdown of some cellular splicing factors indirectly influenced the amounts of other splicing factors [69]. CLIP-seq data shows that binding sites on the HIV-1 genome for specific proteins does not match with the enhancers and suppressors they are thought to bind [70]. The binding data are particularly problematic with hnRNP A1, which has been implicated as a major player in HIV-1 splicing. hnRNP A1 binds RNA both generally and “preferentially” [71]. It has been shown to bind promiscuously across the HIV-1 genome [70], so it is no surprise that HIV-1 studies looking for hnRNP A1 binding sites find them. However, in our own work we did not see the reported effects on splicing with hnRNP A1 knockdown, and in fact, no significant effects at all [59].

Because the SR and hnRNP protein binding motifs are degenerate and fairly ubiquitous, computationally predicted binding sites need to be (but have not always been) backed by actual evidence of their relevance. For example, Esefinder (http://rulai.cshl.edu/cgi-bin/tools/ESE3/esefinder.cgi?process=home) looks for SRSF 1, 2, 5, and 6 binding sites based on SELEX motifs [72]. It finds them abundantly in regions of the HIV-1 genome expected to impact splicing [73,74]. Unfortunately, this search method also finds them equally abundantly in regions not expected to impact splicing, such as within the *gag* intron. As a note of caution, it also finds them equally abundantly in randomly generated sequences [75]. As such, while computational approaches can be helpful, experimental data documenting their validity is essential.

One might reasonably expect that splicing would be impacted by the concentrations and proportions of splicing factors in different cell types. To date, we have found splicing to be very similar across all cell types tested thus far, including both primary cells and cell lines. There is an interesting *tat* transcript difference seen in macrophages, and, as previously noted [76], mouse cells do not support Rev-mediated export and so splice like a Rev mutant (unpublished data in collaboration with the Bieniasz lab). Because cellular splicing also relies on the splicing factors required by HIV-1, it is not possible to tell if a knockdown of a cellular factor inhibits the virus specifically or alters some more general cellular processes.

It is likely that splicing factor binding sites are less like a consensus sequence and more of a combinatorial effect of clustered sequences (see hnRNP H1 binding sites in [70]). hnRNP A1 has been found to dimerize from a starting point and may loop out intronic regions. The effects of such complex interactions may go unappreciated if the experimental focus is exclusively on binding motifs. This is not to say that splicing factors and the sites they bind are not important. When mutated they can cause serious disease, and an SRE can be very easily destroyed [77,78].

In addition to cellular SR and hnRNP splicing proteins, HIV-1 proteins may also feedback into splicing control. In addition to its role as a transcription elongation factor, Tat may promote splicing [79]. As transcription rates are known to affect splicing, these two roles may be connected [80]. Besides Rev’s role in RRE binding and nuclear export discussed above, Rev may also directly interact with hnRNP splicing factors and helicases [81,82]. In addition, Rev may interact with cellular factors to inhibit spliceosome formation [83].

Some potential limitations with HIV-1 splicing work to date are studying splicing acceptors and donors out of context in artificial constructs, low signal to noise ratios in assays, and sub-genomic constructs that do not recapitulate the entirety of HIV-1 splicing in the context of viral infection. Our understanding of splicing factors and their putative binding sites is incomplete and a more thorough comprehension of the roles they play will require greater experimental precision.

HIV-1 RNA secondary structures can also control splicing. Some of the known control elements for A1, A2, and A3 are contained in secondary structures [3,57,84]. Protein binding may depend on RNA secondary structure, or RNA secondary structure may depend on protein binding, or possibly there may be no protein binding involved with these elements—mutations may have changed the secondary structure, and the structure itself may be what controls splicing. We have found that silent mutations to a structure controlling A1 reduced splicing to A1 [3], while mutations that stabilized a structure at A3 reduced splicing to A3 [57]. Thus, the disruption or stabilization of the structure can reduce splicing, depending on the context.

RNA secondary structures within a transcript will change as transcription and splicing proceed, so a structural snapshot of one specific RNA sequence may not represent the dynamics of the ever-changing pre-mRNA transcript. There are many cis-acting splicing regulatory sequences, but the available sites change as transcription and splicing proceed along the genome. The speed of transcription could also change the combination of sequences and structures accessible for splicing or splicing suppression. These possibilities have yet to be adequately explored in the context of HIV-1 splicing regulation.

Finally, RNA epigenetic modifications can also impact splicing [85]. RNA modifications may be a factor in liquid-liquid phase condensates [86], and condensates are implicated in splicing [87]. m(5)C has been shown to regulate HIV-1 splicing [88], and, as the study of epigenomics is a fast-growing field, there may be much more to learn regarding the effects of post-transcriptional modifications on HIV-1 splicing regulation.

## 8. HIV-1 Latency and Splicing—Limited Prospects for a Cure Here

Factors that cause HIV-1 latency are incompletely understood and splicing dysregulation has been implicated as a possible mechanism [89,90,91]. One theory is that low levels of *tat* or *rev* mRNAs play a role in inducing latency [92,93]. If *tat* transcripts were insufficient to produce adequate Tat levels, transcription would abort, and the infected cell would become part of the latent reservoir. Rev is required for export of full-length or partially spliced transcripts. Low levels of Rev would lead to a combined shortage of *gag*/*pro*/*pol* transcripts, *env* transcripts, and genomic RNA, resulting in a nonproductive infection. If low *tat* or *rev* transcript levels induce a state of latency, we would expect that viruses reactivated from the latent reservoir would frequently have mutations and splicing defects affecting *tat* or *rev*.

We examined viruses induced to grow in culture from resting CD4+ T cells taken from two people on suppressive antiviral therapy. The CD4+ T cells used to propagate the outgrowth viruses were analyzed with our deep sequencing splicing assay to quantify the relative amounts of spliced viral transcripts. *tat* and *rev* transcript levels were not reduced in proportion to other spliced transcripts in the outgrowth viruses induced from the latent reservoir, with no loss of splicing efficiency to either splice acceptor A3 (*tat* transcripts) or the A4 acceptor group (*rev* transcripts) [94].

Our findings suggest that the lack of *rev* or *tat* transcripts due to splicing defects is not a primary cause of HIV-1 latency, at least for the subset of viruses that can be induced to grow in culture. It may be that in some cases fluctuations in *tat* or *rev* can lead to transient low transcript levels, and perhaps transcription can be successfully reactivated using Tat or Rev as latency reversing agents for some proviruses. A significant limitation of our own studies to date is that we have only examined inducible proviruses that grew out in culture. There may be a class of proviruses in the 98% that cannot be induced to grow in culture that includes variations of splicing dysregulation. A survey of intact latent proviruses for their ability to regulate splicing, or not, will help resolve this issue.

## 9. Conclusions

Much work has been done on HIV-1 splicing and much remains to be done. The basic mechanisms of some splicing events are still not clear. While some of the splicing regulatory elements are well validated, others are not, and the role of the SR and hnRNP proteins needs more precise clarification. As more and better means of determining RNA secondary structure are developed, more insights into the role of structural elements in splicing and protein interactions will be forthcoming. Although the HIV-1 splice sites are well conserved, a wide range of frequency of usage is tolerated. Splicing from D4 to A7 is stable, possibly mediated by Rev, but by a poorly understood mechanism. The ease with which oversplicing can be induced, with its strongly detrimental effect on the virus, suggests this is an opportunity for therapeutic interventions that target the unique viral mechanisms of splicing suppression.

## Data Availability

The unpublished data presented in this study are not yet publicly available, but are available on request from the corresponding author.
